# Headspace Gas Chromatography Coupled to Mass Spectrometry and Ion Mobility Spectrometry: Classification of Virgin Olive Oils as a Study Case

**DOI:** 10.3390/foods9091288

**Published:** 2020-09-14

**Authors:** María García-Nicolás, Natalia Arroyo-Manzanares, Lourdes Arce, Manuel Hernández-Córdoba, Pilar Viñas

**Affiliations:** 1 Department of Analytical Chemistry, Faculty of Chemistry, University of Murcia, 30100 Murcia, Spain; maria.garcia66@um.es (M.G.-N.); hcordoba@um.es (M.H.-C.); pilarvi@um.es (P.V.); 2 Department of Analytical Chemistry, Faculty of Science, Institute of Fine Chemistry and Nanochemistry, International Agrifood Campus of Excellence (ceiA3), University of Córdoba, 14071 Córdoba, Spain; qa1arjil@uco.es

**Keywords:** gas chromatography, ion mobility spectrometry, mass spectrometry, olive oil classification, headspace, chemometric models

## Abstract

Due to its multiple advantages, ion mobility spectrometry (IMS) is being considered as a complementary technique to mass spectrometry (MS). The goal of this work is to investigate and compare the capacity of IMS and MS in the classification of olive oil according to its quality. For this purpose, two analytical methods based on headspace gas chromatography (HS-GC) coupled with MS or with IMS have been optimized and characterized for the determination of volatile organic compounds from olive oil samples. Both detectors were compared in terms of sensitivity and selectivity, demonstrating that complementary data were obtained and both detectors have proven to be complementary. MS and IMS showed similar selectivity (10 out of 38 compounds were detected by HS-GC-IMS, whereas twelve compounds were detected by HS-GC-MS). However, IMS presented slightly better sensitivity (Limits of quantification (LOQ) ranged between 0.08 and 0.8 µg g^−1^ for HS-GC-IMS, and between 0.2 and 2.1 µg g^−1^ for HS-GC-MS). Finally, the potential of both detectors coupled with HS-GC for classification of olive oil samples depending on its quality was investigated. In this case, similar results were obtained when using both HS-GC-MS and HS-GC-IMS equipment (85.71 % of samples of the external validation set were classified correctly (validation rate)) and, although both techniques were shown to be complementary, data fusion did not improve validation results (80.95% validation rate).

## 1. Introduction

Ion mobility spectrometry (IMS) is a cutting-edge technique that, coupled with gas chromatography (GC), is proving to be a powerful analytical tool in a wide range of research fields, such as foodomics [1,2]. IMS is based on gas phase ion separation inside a drift tube under the influence of a constant electric field at atmospheric pressure. Neutral and gaseous molecules are ionized through the ionization source, and generated ions travel to the drift tube through the shutter grid. The drift time of the ions is characteristic of the analyte because of their different size and shape and is measured in milliseconds (ms). IMS is able to provide analytical information of a great number of samples due to it being a fast and very sensitive technique and, moreover, it requires minimal or no sample preparation; due to its multiple advantages, it is being considered as a complementary technique for mass spectrometry (MS). In this work, both analytical detectors (IMS and MS) are investigated and compared using the characterization of olive oil as a case of study.

According to its quality, olive oil is classified into three different categories: extra virgin (EVOO), virgin (VOO) and lampante (LOO) olive oil. The official method to differentiate between these categories is based on Regulation (EC) 640/2008 of the European Commission and involves physico-chemical analysis together with a sensory assessment by a Panel Test [3]. It is important to take into account that sensory assessment is based on panel tasters and there are few accredited panels in some countries. Moreover, the time required to analyze an olive oil sample, and the high number of tasters necessary, increases the cost of analysis. All of these facts are the main reasons for the search of analytical methods as an alternative or complement to Panel Test analysis.

Olive oil contains a large variety of organic volatile organic compounds (VOCs), which are closely linked to its sensory characteristics and are responsible for its aroma [4]. The determination of VOCs in olive oil can therefore be regarded as a good strategy to classify this high-quality food product. In recent years, several techniques have been reported for this purpose, among them being GC coupled with different sample introduction systems and detectors. Solid phase microextraction (SPME) and GC coupled with MS [5,6,7,8,9,10,11] or flame ionization detectors (FID) [12,13,14,15] have been used for the differentiation of EVOO, VOO, and LOO.

Most of these analytical methods are based on the use of targeted features of VOCs for the differentiation of olive oils, monitoring between 11 and 73 VOCs [5,6,7,8,9]. Although some authors have proposed a strategy based on untargeted fingerprinting combined with chemometric tools [10,11]. In addition, most of the methods propose a binary chemometric model to differentiate between EVOO and non-EVOO samples [8], LOO and non-LOO samples [5,6], or EVOO and VOO samples. The use of two binary models for the discrimination of the three categories has also been explored [7,10]. Sales et al. [11] also proposed a ternary model for the differentiation between the three categories. The chemometric models are based on principal component analysis (PCA), PCA- linear discriminant analysis (LDA), partial least squares-discriminant analysis (PLS-DA) and orthogonal partial least squares discriminant analysis (OPLS-DA).

In addition, two-dimensional GCxGC-MS has demonstrated its analytical advantages in terms of sensitivity, reproducibility, and information potential of the 2D patterns [16]. This method was based on the use of targeted fingerprinting to build a binary model to differentiate between EVOO and non-EVOO also using PCA, PLS-DA, and OPLS-DA. Other analytical methods proposed include metal-oxide sensors (MOS), which have been applied to detect the rancid defect in olive oils [17], and to discriminate edible olive oil (EVOO and VOO) from LOO [18,19].

In recent years, the use of headspace (HS)-GC coupled with IMS [20,21,22,23,24,25,26,27] has also shown great potential as an alternative or complementary strategy to the Panel Test. In the case of GC-IMS, strategies based on targeted [20,24,25,26] and untargeted [20,22,23,24,27] analysis have also been explored. The targeted analysis consisted of the monitoring from two compound (2-hexenal and hexanal or the sum of propanal and 2-propenal) to 15 VOCs. Since a tridimensional map is obtained by GC-IMS, the untargeted analysis consisted of the monitoring of specific untargeted markers or untargeted fingerprinting. The chemometric models were based on the use of PCA, LDA, the k-nearest neighbor method (K-NN), OPLS-DA, hierarchical cluster analysis (HCA), or support Vector Machines (SVM).

As mentioned above, several analytical methods have been proposed for the monitoring of VOCs in olive oil samples and for the classification of olive oil according to its quality, most of them are based on GC-MS and GC-IMS. However, it has not been possible to establish which analytical technique is best for this purpose or if both can be used as alternatives or complementary techniques, since the analytical conditions, the number of samples, and the chemometric procedure are very different in each work. For this reason, the goal of this work is to investigate and compare the capacity of IMS and MS in the classification of olive oil according to its quality. For this purpose, two analytical methods based on HS-GC-MS and HS-GC-IMS have been optimized and characterized for the determination of VOCs. Both methods have been used to analyze 181 olive oil samples of different qualities.

## 2. Materials and Methods

### 2.1. Standards

A total of 38 analytical standards including alcohols (1-octanol, 1-pentanol, 1-hexanol, 2-methyl-1-butanol, 3-methyl-1-butanol, trans-2-hexen-1-ol, cis-2-penten-1-ol, 2-octanol, 1-octen-3-ol), ketones (2-butanone, 2-pentanone, 1-penten-3-one, 2-hexanone, 2-heptanone, 1-octen-3-one, 2-octanone, 2-nonanone, 4-methyl-penan-2-one, 6-methyl-5-hepten-2-one), aldehydes (hexanal, trans-2-hexen-1-al, trans-2-pentenal, decanal, trans-2-decenal, octanal, trans-2-heptenal, heptanal, nonanal, trans-2-octenal), esters (ethyl acetate, ethyl butyrate, ethyl isovalerate, 3-hexenyl acetate, propyl butyrate, hexyl acetate), monoterpenes (limonene), and other compounds such as n-octane and diethyl phthalate, supplied by Sigma-Aldrich Química S.L. (Madrid, Spain) were used for the identification of the characteristic aroma of olive oils.

### 2.2. Samples

A total of 160 olive oil samples (52 EVOO, 56 VOO, and 52 LOO) were analyzed and used for training and evaluation models. In addition, a set of 21 samples (7 EVOO, 7 VOO, and 7 LOO) was used as an external validation set. All samples were from different geographical areas of Spain and were supplied by Sovena S.A. (Sevilla, Spain).

Samples were stored in the freezer in individual bottles without headspace. One gram of these samples was placed in a 20 mL vial closed with a magnetic cap and silicone septum and stored at 4 °C before their analysis (no more than 24 h). Finally, samples were defrosted at room temperature for 30 min, shaken by vortex for 1 min and submitted to HS-GC-MS and HS-GC-IMS analysis.

### 2.3. Instrumentation and Software

Analyses were performed on a multipurpose autosampler (MPS) headspace unit provided by Gerstel and coupled to an Agilent Technologies 6890N (N.05.05 version) gas chromatograph (Agilent, Waldbronn, Germany), which was coupled with a 5973N simple quadrupole mass selective spectrometer equipped with an inert ion source, or with an IMS module from G.A.S (Gesellschaft für Analytische Sensorsysteme mbH, Dortmund, Germany) equipped with a Tritium source and drift tube of 9.8 cm.

In both instruments, analytes were separated in a non-polar GC column HP-5MS UI (Agilent), 30 m length, 0.25 mm internal diameter, and 0.25 µm film thickness.

A vortex from Heathrow Scientific (Heathrow Scientific LLC, Vernon Hills, IL, USA) was also used during the sample treatment. 

MSD Chemstation Data Analysis application, Version G1701EA, revision E.02.02.SP2 was used as software for MS coupling and LAV software (version 2.0.0) from G.A.S for IMS coupling. Data processing was carried out using Matlab (The MathWorks, Natick, MA, USA 2002), PLS Toolbox 5.5 (Eigenvector Research, Inc., Manson, WA, USA), and Statgraphics Centurion XV (StatPoint Technologies Inc., Warrenton, VA, USA).

### 2.4. HS-GC-IMS Analysis

A sample of 1 g was incubated at 90 °C for 3 min (750 rpm). Injection of 750 µL from headspace was carried out using a 2.5 mL syringe at 90 °C in splitless mode. Nitrogen of 99.99% purity (supplied by Air Liquide, Madrid, Spain) was used as the carrier gas at a constant flow rate of 1 mL min^−1^. The oven was set as follows: initial temperature of 50 °C held for 3 min, which was increased from 50 °C to 120 °C at 5 °C min^−1^ and held at 120 °C for 3 min (total run: 20 min). Analytes were driven to the IMS module and ionized by a Tritium source at atmospheric pressure in a positive ion mode. Nitrogen was also used as a drift gas at a constant flow of 150 mL min^−1^. The IMS was operated at a constant voltage of 500 V cm^−1^ and a temperature of 45 °C. Each spectrum was acquired with an average of 32 scans, obtained using a repetition rate of 30 ms, a grid pulse width of 150 μs, and drift and blocking voltages of 241 V and 70 V, respectively.

### 2.5. HS-GC-MS Analysis

A sample of 1 g was incubated at 90 °C for 15 min (750 rpm), no magnetic bar was used for vortexing. Then, 750 µL were injected at 90 °C in splitless mode (using a 2.5 mL syringe). Helium (99.9999% purity) supplied by Air Liquide (Madrid, Spain) was used as the carrier gas at a constant flow rate of 1 mL min^−1^. The oven was set at an initial temperature of 40 °C held for 3 min and heated from 40 °C to 250 °C at a rate of 10 °C min^−1^ and held at 250 °C for 6 min (total run: 30 min). The MS was operated in electron impact mode at 70 eV; ionization energy and data were collected in the range of 20–400 m/z. The ion source, transfer line, and quadrupole temperatures were 230, 300, and 150 °C, respectively. 

### 2.6. Statistical Analysis

As quality control, a standard solution of ketones (octan-2-one, heptan-2-one, hexan-2-one, pentan-2-one, and butan-2-one) at 0.5 mg L^−1^ and one olive oil sample were daily analyzed by both analytical methods.

An HS-GC-IMS analysis results in a tri-dimensional map in which the Y axis represents the retention time in the chromatographic column (in seconds), the X axis represents the drift time in the drift tube (in milliseconds), and the Z axis represents the intensity value (in V) of each compound. For data processing, an initial step of peak alignment was carried out, it was performed only in a retention time scale using a reactant ion peak (RIP) as reference and LAV software. Then, markers were selected by visual exploration of the topographic plots of each sample and their intensities were selected as the analytical signals.

The dataset was formed with all samples and all selected markers. This strategy has been already proposed for IMS data processing, specifically for olive oil analysis [20,22,23], although, it has been also used for other food applications [1].

Data from HS-GC-MS were processed following two different strategies: the use of the whole chromatographic profile, i.e., the total ion chromatogram (TIC), or the use of the areas of the main chromatograph peaks. No data pre-processing was needed for the dataset of peak integration. However, in the case of the TIC dataset, baseline correction was necessary. It was corrected by subtracting the mean value of the background.

After data pre-processing, the tree different datasets were divided into two groups: the training set for the construction of the chemometric models (128 samples) and evaluation set validation (32 samples) for the optimization of method parameters. In addition, an external test set (21 samples) was used for method validation. The constructions of chemometric models were carried out based on previous reported methods for the classification of olive oil [20,22]. A non-supervised PCA analysis using auto-scales was carried out in order to reduce the dimensionality. Then, PCA scores were used to carry out an LDA. Finally, k-NN, using k = 3, was applied to classify the samples.

## 3. Results

### 3.1. Optimization of HS-GC-MS and HS-GC-IMS Methods

The optimization of both analytical methods was carried out with the aim of achieving the best results in terms of intensity and separation between peaks, from the previously described conditions [6,7,8,9,10,20,21,22,23]. With this purpose, the variable injection volume, time, and temperature of incubation, and injector temperature were investigated using both techniques.

The effect of the injection volume was studied between 500 µL and 750 µL, obtaining higher intensity signals with 750 µL. The effect of the sample incubation temperature was studied between 60 °C and 90 °C. High temperatures facilitate the release of volatile organic compounds with high boiling points. This caused an increase in the number of signals and their intensities in the 60–90 °C range, therefore, 90 °C was selected as the optimum. Then, the sample incubation time was studied between 5 and 20 min. For HS-GC-MS, the signal intensities increased as the incubation time increases, however no significant differences were found between 15 and 20 min, and therefore, sample incubation time was set at 15 min (Supplementary Materials Figure S1). In the case of HS-GC-IMS, no significant differences were appreciated between 5 and 20 min, thus the incubation time was investigated in the range of 1 to 5 min, selecting 3 min as the optimum, since higher temperatures did not improve the spectrum (Supplementary Materials Figure S2). Finally, the injector temperature was studied between 70 °C and 90 °C and no significant differences were found. For this reason, the injector temperature was set to 90 °C (temperature of sample incubation). Based on previous work, the salt addition was not considered since it does not cause any increase in VOC signals and in contrast, a reduction of some signals is observed when the saturated salt solution is added to the olive oil [23].

The oven program was also studied for both methods in order to achieve optimal conditions. The best peak separation in HS-GC-MS was obtained with the following conditions: initial temperature of 40 °C held for 3 min, increased to 250 °C at 10 °C min^−1^ and held at 250 °C for 6 min. For HS-GC-IMS, temperatures higher than 120 °C were not recommended by the manufacturer of the IMS, since the drift tube has a temperature limitation of 100 °C, therefore the oven was set as follows: initial temperature of 50 °C were held for 3 min, which was increased from 50 °C to 120 °C at 5 °C min^−1^ and held at 120 °C for 3 min.

Finally, the drift tube temperature of HS-GC-IMS was investigated between 45 and 75 °C and no significant differences were obtained, therefore 45 °C was selected for further experiments.

### 3.2. Identification and Quantification of Volatile Compounds in Olive Oil Samples by HS-GC-MS and HS-GC-IMS

**Figure 1:**
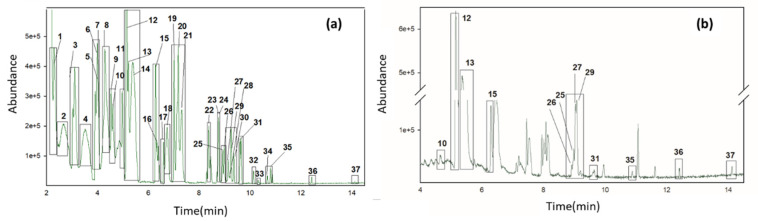
Total ion chromatogram (TIC) of a standards mixture (**a**) and an extra virgin olive oil (EVOO) sample (**b**) showing the volatile compounds identified by headspace gas chromatography (HS-GC) coupled with mass spectrometry (MS): (1) ethyl acetate, (2) 2-butanone, (3) 2-pentanone, (4) 1-penten-3-one, (5) 2-methyl-1-butanol, (6) 3-methyl-1-butanol, (7) 4-methyl-pentan-2-one, (8) trans-2-pentenal, (9) 1-pentanol, (10) cis-2-penten-1-ol, (11) 2-hexanone, (12) n-octane, (13) hexanal, (14) ethyl butyrate, (15) trans-2-hexen-1-al, (16) ethyl isovalerate, (17) 1-hexanol, (18) trans-2-hexen-1-ol, (19) 2-heptanone, (20) propyl butyrate, (21) heptanal, (22) trans-2-heptenal, (23) 1-octen-3-one, (24) 1-octen-3-ol, (25) 6-methyl-5-hepten-2-one, (26) 2-octanone, (27) 2-octanol, (28) octanal, (29) 3-hexenyl-acetate, (30) hexyl acetate, (31) limonene, (32) trans-2-octenal, (33) 1-octanol, (34) 2-nonanone, (35) nonanal, (36) decanal, (37) trans-2-decenal, (38) diethyl phthalate.

**Figure 2:**
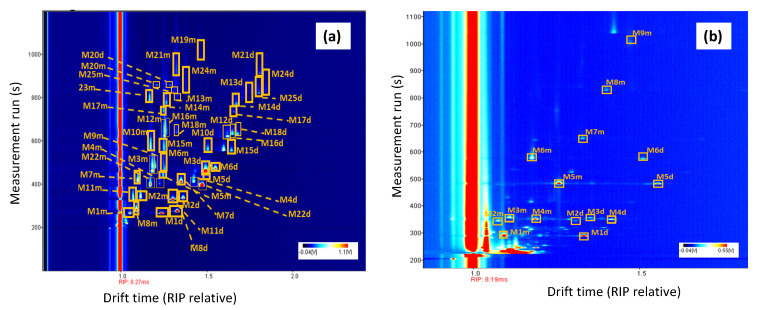
Topographic maps of the standards mixture (**a**) and an EVOO sample (**b**) showing the volatile compounds identified by HS-GC coupled with ion mobility spectrometry (IMS): (M1m) 2-butanone monomer, (M1d) 2-butanone dimer, (M2m) 2-pentanone monomer, (M2d) 2-pentanone dimer, (M3m) 2-hexanone monomer, (M3d) 2-hexanone dimer, (M4m) 2-methyl-1-butanol monomer, (M4d) 2-methyl-1-butanol dimer, (M5m) 1-pentanol monomer, (M5d) 1-pentanol dimer, (M6m) hexanal monomer, (M6d) hexanal dimer, (M7m) trans-2-pentenal monomer, (M7d) trans-2-pentenal dimer, (M8m) ethyl acetate monomer, (M8d) ethyl acetate dimer, (M9m) ethyl butyrate monomer, (M10m) trans-2-hexen-1-al monomer, (M10d) trans-2-hexen-1-al dimer, (M11m) 1-penten-3-one monomer, M11d) 1-penten-3-one dimer, (M12m) 2-heptanone monomer, (M12d) 2-heptanone dimer, (M13m) 2-octanone monomer, (M13d) 2-octanone dimer, (M14m) 1-octen-3-one monomer, (M14d) 1-octen-3-one dimer, (M15m) ethyl isovalerate monomer, (M15d) ethyl isovalerate dimer, (M16m) propyl butyrate monomer, (M16d) propyl butyrate dimer, (M17m) trans-2-heptenal monomer, (M17d) trans-2-heptenal dimer, (M18m) heptanal monomer, (M18d) heptanal dimer, (M19m) nonanal monomer, (M20m) limonene monomer, (M20d) limonene dimer, (M21m) trans-2-octenal monomer, (M21d) trans-2-octenal dimer, (M22m) 4-methyl-pentan-2-one monomer, (M22d) 4-methyl-pentan-2-one dimer, (M23m) 6-methyl-5-hepten-2-one monomer, (M24m) hexyl acetate monomer, (M24d) hexyl acetate dimer, (M25m) 3-hexenyl acetate, (M25d) 3-hexenyl acetate.

A total of 160 olive oil samples of different qualities (52 EVOO, 56 VOO, and 52 LOO) were analyzed with both analytical techniques and an attempt was then made to identify as many signals as possible. For this purpose, 38 standard compounds that have been previously described in olive oil samples and cited in Section 2.1, were prepared at 2 µg g^−1^ in refined oil and also analyzed for both methods. Standard compound information is shown in Supplementary Materials Table S1. All compounds could be detected in a mixture using the HS-GC-MS method (Figure 1a), however only 25 of the analyzed compounds could be detected by HS-GC-IMS (Figure 2a). Hence, 13 VOCs that were monitored by the HS-GC-MS device, were not detected under the developed HS-GC-IMS method, these compounds are: 1-hexanol, 3-methyl-1-butanol, 2-nonanone, decanal, trans-2-decenal, trans-2-hexen-1-ol, 1-octen-3-ol, octanal, cis-2-penten-1-ol, 2-octanol, 1-octanol, n-octane, and diethyl phthalate.

In the olive oil samples analyzed by HS-GC-IMS, a total of 10 VOCs were identified, named 2-pentanone, hexanal, ethyl acetate, trans-2-hexen-1-al,1-penten-3-one, heptanal, nonanal, 4-methyl-pentan-2-one, hexyl acetate, and trans-2-pentenal. In the case of HS-GC-MS, 12 VOCs could be identified in the analyzed samples using the extracted ion chromatogram (EIC): hexanal, trans-2-hexen-1-al, 6-methyl-5-hepten-2-one, cis-2-penten-1-ol, n-octane, 2-octanone, 2-octanol, limonene, 3-hexenyl acetate, nonanal, decanal, and trans-2-decenal. The identification of compounds in HS-GC-MS was confirmed using the real standards and the match with the library. However, the data of the libraries available for IMS are very limited, and in this case, compounds were identified only by comparison of retention time and drift time with the real standards. Figure 2b shows an olive oil spectrum obtained by HS-GC-IMS with 9 of 10 VOCs identified, where the monomers and dimers of each compound have been indicated. No olive oil samples were found showing all identified compounds. Figure 1b shows the HS-GC-MS chromatogram of an olive oil sample with the 12 VOCs identified.

In order to evaluate both analytical methods in term of validation, calibration curves were established for ethyl acetate, 1-penten-3-one, 2-pentanone, 4-methyl-pentan-2-one, hexanal, trans-2-pentenal, trans-2-hexen-1-al, heptanal, 6-methyl-5-hepten-2-one, 3-hexenyl acetate, nonanal, decanal, trans-2-decenal, and hexyl acetate using refined oil spiked at six concentration levels between 0.05 and 50 µg g^−1^. Each concentration level was injected twice. The statistical parameters were calculated by least-square regression for HS-GC-MS. In the case of HS-GC-IMS data, because each compound can generate two signals corresponding to monomers and dimers, different calibration graphs were considered. Specifically, the least-square and logarithmic regressions were tested. In both cases, graphics were constructed using the signal of the monomer and the dimer or the sum of both monomer and dimer signals. The best results were obtained using the logarithmic regressions and the sum of monomer and dimer signals. As can be seen in Table 1, satisfactory determination coefficients were obtained in all the cases, although they were slightly better in MS (R^2^ > 0.98 for MS and R^2^ > 0.92 for IMS).

Limits of detection (LODs) and quantification (LOQs) were estimated as 3 x signal-to-noise ratio (S/N) and 10 × S/N, respectively. The LOQs ranged between 0.2 and 2.1 µg g^−1^ for HS-GC-MS and between 0.08 and 0.82 µg g^−1^ for HS-GC-IMS. Trans-2-hexen-1-al, hexanal, and nonanal could be detected and quantified for both methods, although slightly better LOQ were obtained with HS-GC-IMS.

In order to compare the two different analytical methods, a simple regression using Statgraphic software was carried out. This study was performed with the compound that could be detected and quantified by both techniques, named trans-2-hexen-1-al, hexanal, and nonanal. In all the cases, P-value was greater than 0.05, so there was not a statistically significant relationship between IMS and MS data at the 95.0% confidence level. This could be justified by the difference in the analytical response obtained since, at the range of concentrations studied, MS data were adjusted to a linear regression while a logarithmic adjustment was necessary with the IMS data.

Calibration curves were used to quantify the identified compounds by both techniques in the olive oil samples and this information was used to compare the three categories applying ANOVA and Tukey’s test as post hoc test. Table 2 shows the concentration average of each analyte for each category and the Tukey’s test results using both techniques.

The results showed that the compounds heptanal, 6-methyl-5-hepten-2-one, nonanal, and trans-2-decenal allow differentiation between LOO and the edible olive oil samples. All these compounds have been defined as descriptors of rancid, fatty, or mustiness-humidity [28,29,30]. Otherwise, ethyl acetate, 3-hexenyl acetate, and trans-2-hexen-1-al enable the differentiation between non-defective (EVOO) and defective (non-EVOO) olive oil samples, due to these compounds being associated with fruity, aromatic, green, or sweet sensory descriptors [23,31]. Due to the differences in sensitivity and precision of both methods, the hexanal concentration obtained by HS-GC-IMS allowed the differentiation of EVOO from the rest of the categories, while this compound enabled discrimination of LOO samples using HS-GC-MS. Depending on its concentration, hexanal has been related to a mustiness-humidity, fusty, winey-vinegary, or rancid defect, but also to green-sweet, green apple and grass sensory descriptors [20,22,23,24,32,33,34,35]. The ketone 1-penten-3-one was the only compound that allowed the differentiation between the three categories, which has previously been described as an accurate marker of EVOO quality [36]. This compound is related to green, pungent, and sweet sensory descriptors [20,22,23,32,33,34,35]. No significant differences were found for 2-pentanone, 4-methyl-pentan-2-one, related with sweet, fruity or ethereal sensory properties [22,32], and decanal, related to rancid defect [20,23]. The trans-2-pentenal, which is related to a winey-vinegary, pungent, green characteristic [20,23,34,35], enabled the observation of differences between EVOO and LOO, although, VOO cannot be distinguished from EVOO or LOO.

As has been shown, certain compounds can be associated with a particular category. However, the high variability observed within the same group did not allow the establishment of a compound concentration limit in each category and therefore chemometric models are necessary.

### 3.3. Chemometrics for Olive Oil Classification According to Its Quality

Chemometric models were constructed using the data obtained by HS-GC-MS and HS-GC-IMS. Different chemometric approaches were investigated to process HS-GC-MS data, specifically, the selection of significant marker peaks (known and unknown compounds) and the use of the total ion chromatogram (TIC).

As described in Section 2.6, HS-GC-IMS data processing has been well-studied and it was performed following the indication of Contreras et al. [23] In this case, chemometric models based on PCA-LDA were preferred to the use of modelling techniques such as SIMCA or QDA. Several papers have demonstrated the poor performance of SIMCA as compared to other methods, e.g., to LDA. The fact that LDA was developed by statisticians, whereas SIMCA was developed by chemists (chemometricians) might contribute to the characteristic differences between their theoretical backgrounds. For example, SIMCA does not require any distributional assumptions, whereas LDA assumes normal distribution and equal variances for each class [37]. In addition, Nikita et al. observed that QDA does not give better results than LDA and does not offer an alternative to LDA [38].

**Figure 3:**
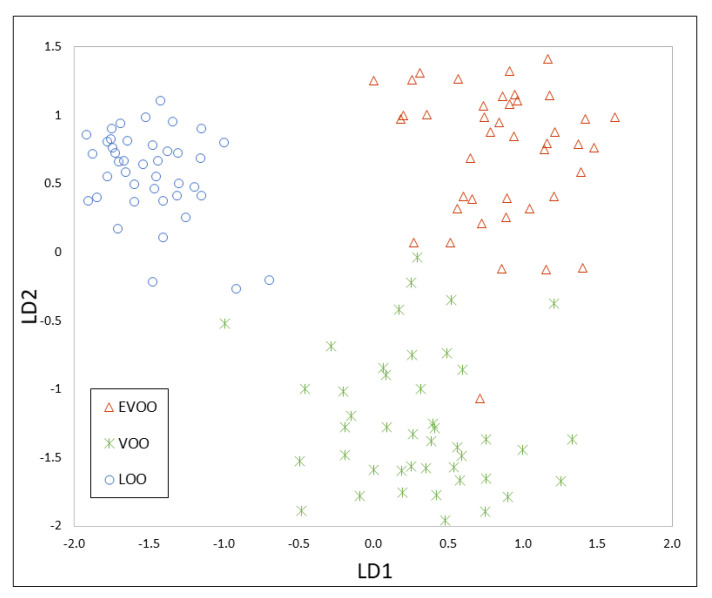
Principal component analysis- linear discriminant analysis (PCA-LDA) model constructed using the HS-GC-IMS markers.

A total of 86 markers were selected by visual exploration of the topographic plots obtained by HS-GC-IMS and their intensities were used as a dataset. Therefore, the data matrix had a dimension of 160 (samples) × 86 (markers). This matrix was split into two datasets: calibration samples (80%) and evaluation samples (20%), i.e., 128 samples were used for model construction and 32 for the optimization of K in the K-NN model. After PCA, 29 principal components were obtained including the 99.07% of variance cumulative. The results of PCA are shown in Supplementary Materials Figure S3. The PCA-LDA model showed a clear separation between each group of samples (Figure 3).

The k-NN was then optimized using the evaluation set. A classification rate of 87.50% and 84.50% were obtained for k = 3 and k = 5, respectively, so k = 3 was selected as optimum. Finally, the chemometric models were applied to classify other 20 samples (external validation set), obtaining a validation rate (percentage of samples of the external validation set classified correctly) of 85.71% owing to three samples being incorrectly classified: one EVOO as VOO, one VOO as EVOO, and one VOO as LOO (Table 3).

As mentioned above, two different chemometric strategies were investigated to process HS-GC-MS data. The first strategy consisted of using the chromatographic peak areas, including known and unknown compounds, in order to obtain the maximum possible information of the chromatogram. To do so, a total of 95 peaks were integrated and processed. The second strategy consisted of the use of the TIC and, firstly, the need for an alignment step was evaluated; no misalignment was detected between samples. However, a baseline shift was observed, and therefore a pre-processing step consisting of a baseline correction was carried out. Baseline was corrected by subtracting the mean value of background (an empty section of peaks, between 14.9 and 15.34 min). In this case, the dimensions of the data matrix were 160 × 95 for the first strategy and 160 × 5272 for the second strategy, since TIC was composed of 5272 values.

**Figure 4:**
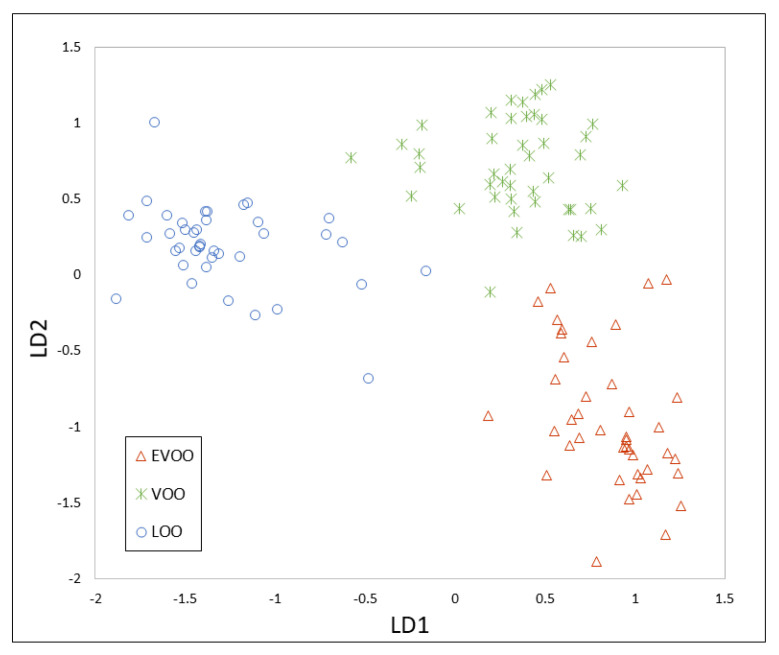
PCA-LDA model constructed using the peak integration of the HS-GC-MS method.

**Figure 5:**
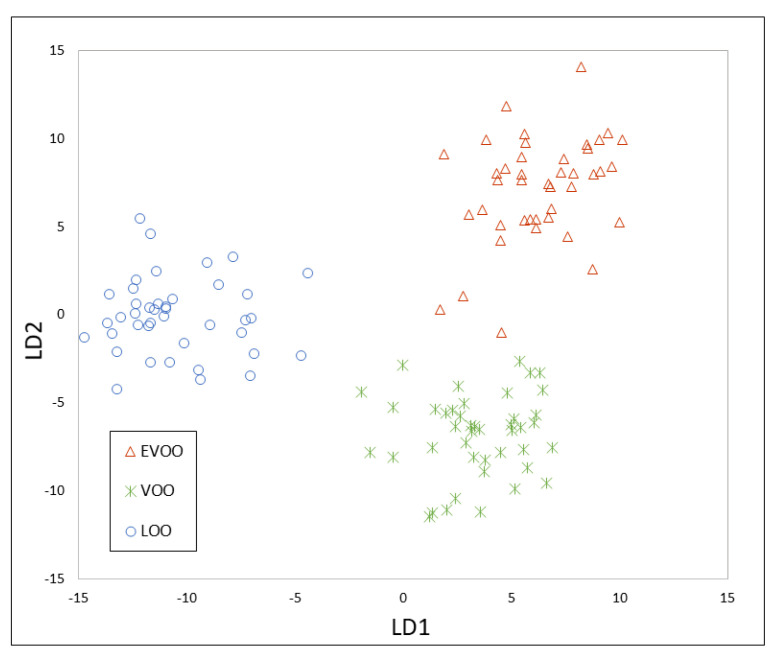
PCA-LDA model constructed using the TIC of the HS-GC-MS method.

These matrices were also split into two datasets: a training set (128 samples) and an evaluation set (32 samples). The PCA allowed a reduction of the dimensionality to 58 and 122 principal components (99% variance cumulative) for the first and second strategy, respectively. The results of the PCA are shown in Supplementary Materials Figures S4 and S5. PCA-LDA models are shown in Figure 4 and Figure 5.

Different K-NN models, using k = 3 and k = 5, were applied to the evaluation set. The same classification rate was obtained for peak integration data (78.13%), however better results were obtained with k = 3 for TIC data (87.50% for k = 3 and 84.40 % for k = 5). The application of the k-NN method (k = 3) to the external validation set is shown in Table 4 and Table 5, and as can be seen, better validation rates (85.71%) were obtained using the TIC, similar to that obtained by IMS. In this case, all LOO samples were also correctly classified. However, one EVOO and two VOO were also misclassified. The results obtained with only one chemometric model are comparable to those previously reported by means of two sequential models [10]. The use of the chromatographic peak areas showed validation rates of 76.19%, where two EVOO samples were classified as VOO, two VOO samples as EVOO and LOO, and one LOO sample as VOO.

### 3.4. Data Fusion of MS and IMS

HS-GC-MS and HS-GC-IMS showed the same validation rate, demonstrating that both techniques are appropriate for the classification of olive oil samples. In addition, they have been shown to be complementary, since they allowed the detection and quantification of different compounds. Therefore, in an attempt to improve the validation rate, the construction of chemometric models using data fusion was investigated.

**Figure 6:**
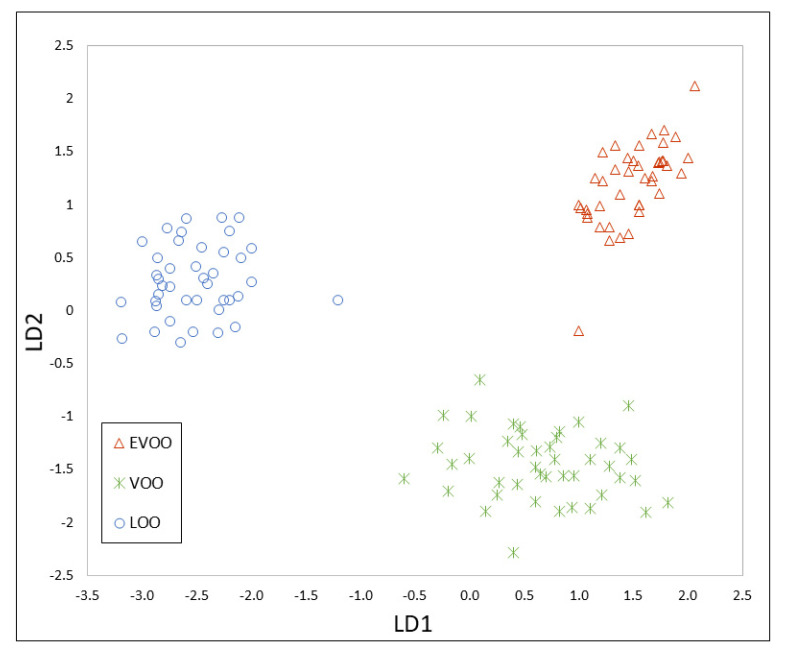
PCA-LDA model constructed using data fusion.

With that purpose, the 95 peak areas selected from HS-GC-MS data and the 86 markers from topographic maps of HS-GC-IMS were united to characterize each olive oil sample, i.e., a final data matrix with dimensions of 160 (samples) × 181 (markers) was used to carry out the chemometric treatment. After PCA, the dataset was decreased to 45 principal components (99.10% variance cumulative). Better results were obtained using k = 3 when the K-NN method was applied to the evaluation set (k = 3, 81.25%; k = 5, 71.85%), so k = 3 was also selected as optimum. A validation success rate of 80.95% (Table 6) was obtained despite the good separation obtained with PCA-LDA (Figure 6). One EVOO sample was classified as VOO and one VOO was upgraded to EVOO. The most important mistake was made when classifying the LOO sample, since one of them was classified as VOO and the other one as EVOO. For all these reasons, data fusion did not improve the results obtained and, in this case, HS-GC-MS and HS-GC-IMS could not be used as complementary techniques, but rather as alternatives.

## 4. Discussion

In this work, two coupling techniques, HS-GC-MS and HS-GC-IMS, have been evaluated for the classification of olive oil samples.

Both techniques have proven to be complementary for identification and quantification of characteristic olive oil VOCs, since they were able to monitor different compounds. Specifically, ten compounds were identified in olive oil samples using HS-GC-IMS, whereas twelve compounds were identified using HS-GC-MS. Only trans-2-hexen-1-al, hexanal, and nonanal were identified by both techniques. Regarding sensitivity, HS-GC-IMS showed LOQ slightly better.

To obtain calibration curves, a least-square regression was applied in HS-GC-MS; however, logarithmic regressions considering the sum of monomer and dimer signals as analytical responses had to be used in HS-GC-IMS. Satisfactory determination coefficients were obtained in all cases, although they were slightly better in MS.

Certain compounds detected for IMS and/or MS techniques can be associated with a particular category. Specifically, heptanal, 6-methyl-5-hepten-2-one, nonanal, and trans-2-decenal allowed differentiation between LOO and the edible olive oil samples; whereas ethyl acetate, 3-hexenyl acetate, and trans-2-hexen-1-al enabled the differentiation between non-defective (EVOO) and defective (non-EVOO) olive oil samples in concordance with Romero et al. [5] The ketone 1-penten-3-one was the only compound that allowed the differentiation between the three categories, also in concordance with Garrido-Delgado et al. [36]. However, the high variability observed within the same group did not allow the establishment of a compound concentration limit in each category, making necessary the use of chemometric models.

Finally, HS-GC-MS and HS-GC-IMS models showed the same validation rate (85.71%) for classification of olive oil samples, although it was necessary to use the entire chromatographic profile obtained by MS to achieve the results obtained by IMS. The validation results of HS-GC-MS are similar to other previous reported work, although in those cases, an SPME step was used instead of HS. Quintanilla-Casas et al. obtained results of 89.2% using two sequential PLS discriminant analysis models and an untargeted fingerprinting strategy [10]. Sales et al. obtained 70 and 85% of classification rate monitoring 15 VOCs [7] or using untargeted fingerprinting strategies, respectively [11]. The other work proposed for olive oil classification do not distinguish between the three classes of olive oil so they could not be compared. In the case of HS-GC-IMS, classification rates ranged between 94% and 100% using a 60 m capillary column, ramped temperature, and a supervised method such as OPLS-DA [23]. Gerhardt et al. obtained 83.3% using untargeted fingerprinting and LDA chemometric models [27] and Valli et al. obtained 77% using a features signal of 15 VOCs and PCA and PLS-DA models [25].

Therefore, both techniques are appropriate for the classification of olive oil samples and could be used as complementary or screening support to the test panel. However, both techniques should not be applied together since data fusion did not improve the results.

## Figures and Tables

**Table 1:** Calibration curves and performance characteristics of the headspace-gas chromatography (HS-GC) coupled with mass spectrometry (MS) and ion mobility spectrometry (IMS) methods.

	HS-GC-MS				HS-GC-IMS					
Analyte	RT (min)	Linear Dynamic Range (µg g^−1^)	R^2^	LOQ	RT (s)	Drift Time Monomer (ms)	Drift Time Dimer (ms)	Logarithmic Dynamic Range (µg g^−1^)	R^2^	LOQ
Ethyl acetate	-	-	-	-	276.2	9.0	11.0	0.08–20	0.985	0.08
1-penten-3-one	-	-	-	-	328.7	8.8	10.7	0.08–20	0.989	0.08
2-pentanone	-	-	-	-	334.6	9.2	11.3	0.15–50	0.993	0.15
4-methyl-pentan-2-one	-	-	-	-	396.9	9.6	12.2	0.10–50	0.990	0.10
Hexanal	5.2	0.90–50	0.987	0.90	462.3	10.3	12.8	0.75–50	0.999	0.75
Trans-2-pentenal	-	-	-	-	411.8	9.1	11.2	0.82–50	0.993	0.82
Trans-2-hexen-1-al	6.3	0.38–50	0.995	0.38	554.4	9.6	12.4	0.15–50	0.984	0.15
Heptanal	-	-	-	-	631.6	10.8	13.8	0.42–50	0.976	0.42
6-methyl-5-hepten-2-one	8.9	0.44–50	0.993	0.44	-	-	-	-	-	-
3-hexenyl acetate	9.2	0.48–50	0.995	0.48	-	-	-	-	-	-
Nonanal	10.8	0.20–50	0.996	0.20	989.0	12.0	ND	0.10–50	0.920	0.10
Decanal	12.4	1.55–50	0.996	1.55	-	-	-	-	-	-
Trans-2-decenal	14.4	2.10–50	0.985	2.10	-	-	-	-	-	-
Hexyl acetate	ND	ND	ND	ND	830.6	11.3	15.5	0.21–20	0.993	0.21

Calibration curves only were constructed for the compounds identified in olive oil samples. RT: Retention time, LOQ: Limits of quantification.

**Table 2:** Average concentrations (µg·g^−1^) and standard deviation of each analyte including results of the Tukey’s test.

	HS-GC-MS			HS-GC-IMS			Sensory Properties [20,21,22,23,24,32,33,34,35]
Analyte	EVOO	VOO	LOO	EVOO	VOO	LOO	
Ethyl acetate	-	-	-	0.17 ± 0.16 ^a^	0.46 ± 0.56 ^b^	0.99 ± 1.47 ^b^	Fusty, winey-vinegary, fruity, aromatic, ethereal, sweet
1-penten-3-one	-	-	-	0.66 ± 0.09 ^a^	0.31 ± 0.01 ^b^	0.14 ± 0.01 ^c^	Green, pungent, sweet
2-pentanone	-	-	-	0.19 ± 0.14 ^a^	0.17 ± 0.08 ^a^	0.15 ± 0.09 ^a^	Sweet
4-methyl-pentan-2-one	-	-	-	0.70 ± 0.71 ^a^	0.62 ± 0.49 ^a^	1.52 ± 0.77 ^a^	Fruity, sweet, ethereal
Hexanal	0.70 ± 0.33 ^a^	0.91 ± 0.22 ^a^	1.10 ± 0.4 ^b^	0.86 ± 0.01 ^a^	0.97 ± 0.60 ^b^	0.99 ± 0.20 ^b^	Mustiness-humidity, fusty, winey-vinegary, rancid, green-sweet, green apple, grass
Trans-2-pentenal	-	-	-	0.83 ± 0.12 ^a^	1.06 ± 0.12 ^a,b^	1.30 ± 0.13 ^b^	Winey-vinegary, pungent, green
Trans-2-hexen-1-al	0.70 ± 0.28 ^a^	0.45 ± 0.17 ^b^	0.40 ± 0.19 ^b^	0.74 ± 0.15 ^a^	0.48 ± 0.19 ^b^	0.39 ± 0.21 ^b^	Mustiness-humidity, fusty, winey-vinegary, rancid, bitter almond, green
Heptanal	-	-	-	0.49 ± 0.10 ^a^	0.50 ± 0.08 ^a^	0.94 ± 0.12 ^b^	Rancid, fatty, woody
6-methyl-5-hepten-2-one	0.16 ± 0.15 ^a^	0.18 ± 0.29 ^a^	0.67 ± 0.93 ^b^	-	-	-	Mustiness-humidity, fusty, rancid, pungent, green
3-hexenyl acetate	1.01 ± 0.17 ^a^	0.50 ± 0.23 ^b^	0.47 ± 0.3 ^b^	-	-	-	Green banana, green leaves, fruity
Nonanal	0.28 ± 0.12 ^a^	0.31 ± 0.18 ^a^	0.63 ± 0.36 ^b^	0.35 ± 0.15 ^a^	0.37 ± 0.19 ^a^	0.76 ± 0.42 ^b^	Rancid, fatty, waxy, pungent
Decanal	1.56 ± 0.20 ^a^	1.63 ± 0.75 ^a^	1.63 ± 0.53 ^a^	-	-	-	Rancid
Trans-2-decenal	2.17 ± 1.97 ^a^	2.32 ± 1.39 ^a^	2.41 ± 1.60 ^b^	-	-	-	Rancid
Hexyl acetate	-	-	-	0.52 ± 0.01^a^	0.47 ± 0.01 ^a^	0.22 ± 0.09 ^b^	Fruity, green, sweet

a, b, c superscripts represent different groups of classification for a specific compound according to the Tukey’s test. EVOO: extra virgin olive oil; VOO: virgin olive oil; LOO: lampante olive oil.

**Table 3:** Validation matrix by k-NN using HS-GC-IMS data.

		Actual Classes			
		EVOO	VOO	LOO	
**Predicted classes**	EVOO	6	1	0	**Success 85.71%**
	VOO	1	5	0	
	LOO	0	1	7	

**Table 4:** Validation matrix by k-NN using chromatographic peak areas obtained with HS-GC-MS.

		Actual Classes			
		EVOO	VOO	LOO	
**Predicted classes**	EVOO	5	1	0	**Success 76.19%**
	VOO	2	5	1	
	LOO	0	1	6	

**Table 5:** Validation matrix by k-NN using the TIC obtained with HS-GC-MS.

		Actual Classes			
		EVOO	VOO	LOO	
**Predicted classes**	EVOO	6	1	0	**Success 85.71%**
	VOO	1	5	0	
	LOO	0	1	7	

**Table 6:** Validation matrix by k-NN using data fusion of HS-GC-MS and HS-GC-IMS.

		Actual Classes			
		EVOO	VOO	LOO	
**Predicted classes**	EVOO	6	1	1	**Success 80.95%**
	VOO	1	6	1	
	LOO	0	0	5	

## Supplementary Materials

The following are available online at https://www.mdpi.com/2304-8158/9/9/1288/s1, Figure S1: HS-GC-MS chromatogram obtained for 5, 10, 15 y 20 min of incubation time, Figure S2. HS-GC-IMS spectra obtained for 1, 3 y 5 min of incubation time, Figure S3. PCA scores and loading plot using the HS-GC-IMS markers, Figure S4. PCA scores and loading plot using the peak integration of the HS-GC-MS method, Figure S5. PCA scores and loading plot using the TIC of the HS-GC-MS method Table S1. Chemical information of the VOCs.
