# Supplementary material for: Headspace Gas Chromatography Coupled to Mass Spectrometry and Ion Mobility Spectrometry: Classification of Virgin Olive Oils as a Study Case
Source: Foods. 2020 Sep 14;9(9):1288. doi: 10.3390/foods9091288 (PMC7555980; doi:10.3390/foods9091288)
Supplement: Supplementary file 1 [file foods-09-01288-s001.pdf]

*foods*

MDPI

*Supplementary Material*

# Headspace gas chromatography coupled to mass spectrometry and ion mobility spectrometry: Classification of virgin olive oils as a study case

**María García-Nicolás[1], Natalia Arroyo-Manzanares[1*], Lourdes Arce[2], Manuel Hernández-Córdoba[1], Pilar Viñas[1]**

[1] Dept. Analytical Chemistry, Faculty of Chemistry, University of Murcia, Murcia, Spain; maria.garcia66@um.es (M.G.-N.); hcordoba@um.es (M.H.-C.); pilarvi@um.es (P.V.)

[2] Dept. Analytical Chemistry, Faculty of Science, Institute of Fine Chemistry and Nanochemistry. International Agrifood Campus of Excellence (ceiA3), University of Córdoba, Córdoba, Spain; qa1arjil@uco.es (L.A.)

* Correspondence: natalia.arroyo@um.es (N.A.-M.)

Received: date; Accepted: date; Published: date

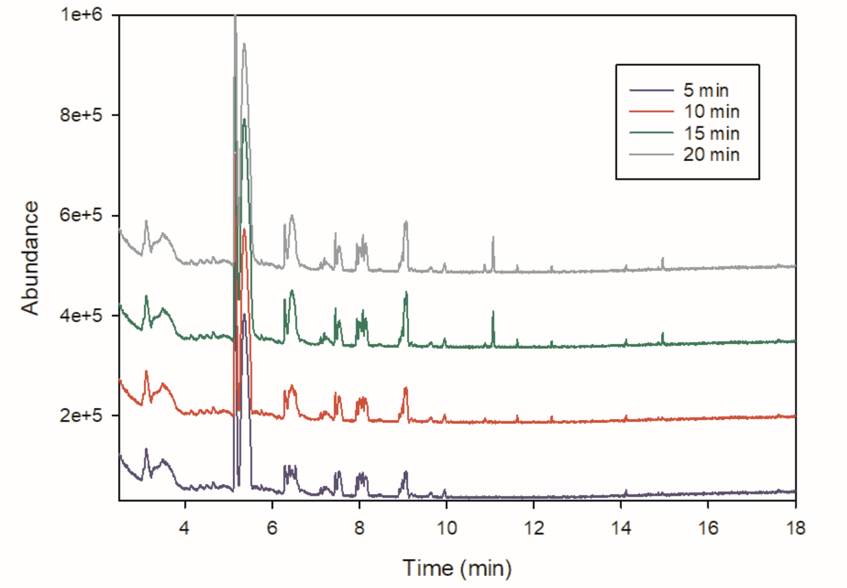


**Supplemental Figure S1**. HS-GC-MS chromatogram obtained for 5, 10, 15 y 20 minutes of incubation time

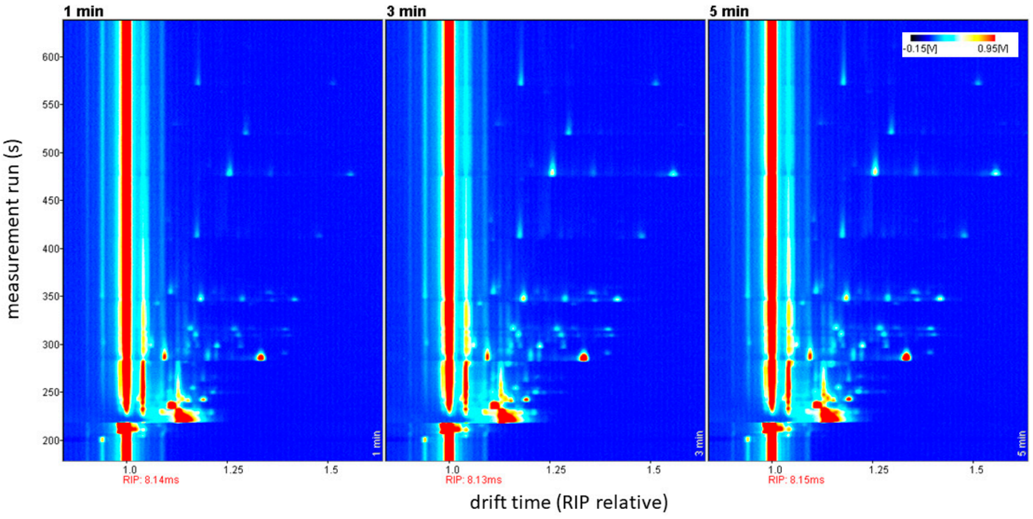


**Supplemental Figure S2**. HS-GC-IMS spectra obtained for 1, 3 y 5 minutes of incubation time

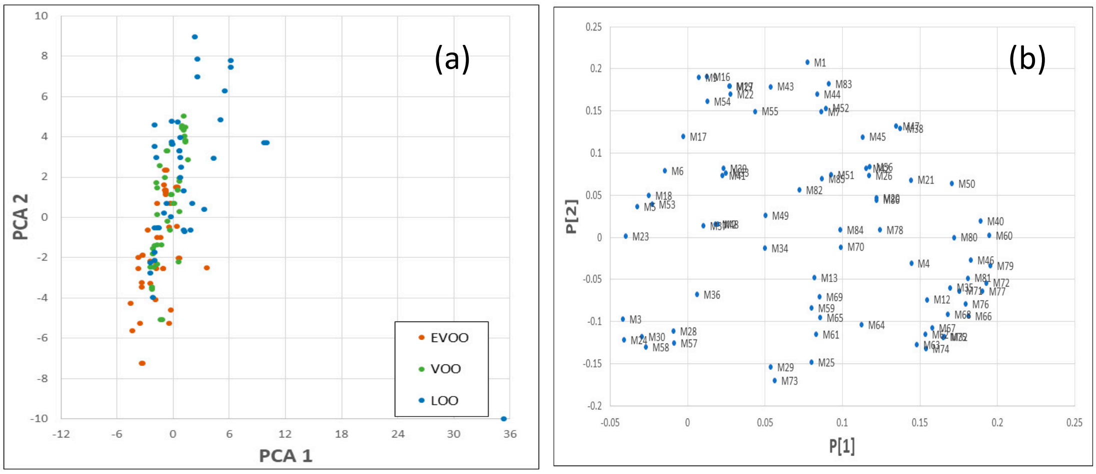


**Supplemental Figure S3**. PCA scores and loading plot using the HS-GC-IMS markers

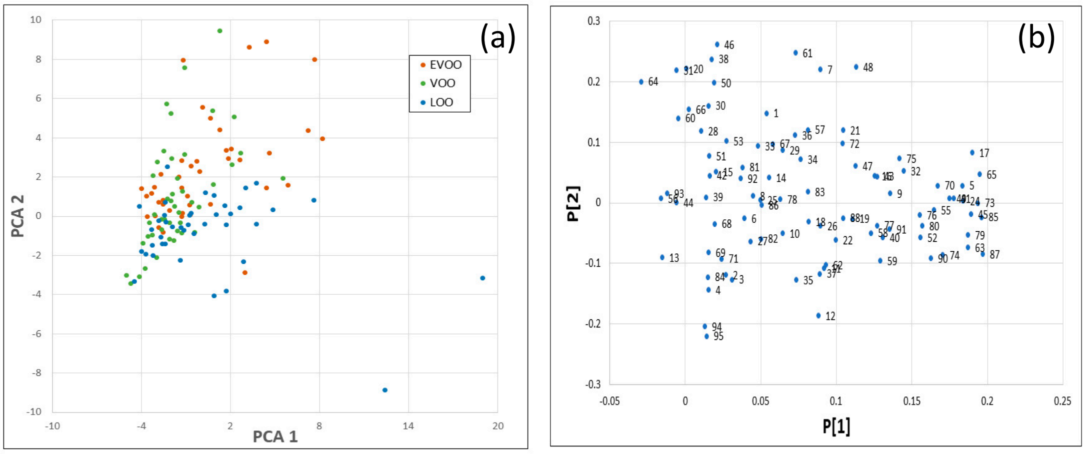


**Supplemental Figure S4**. PCA scores and loading plot using the peak integration of the HS-GC-MS method

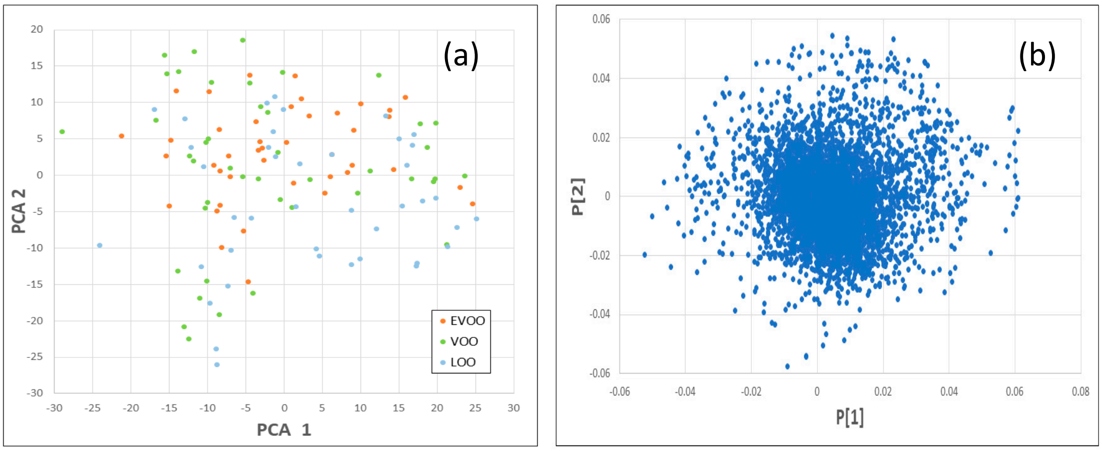


**Supplemental Figure S5**. PCA scores and loading plot using the TIC of the HS-GC-MS method.

foods

MDPI

**Supplemental Table S1**. Chemical information of the VOCs monitored

| | | Molecular formula | Molecular weight | *m/z* theoretical ions |
|---|---|---|---|---|
| Alcohols | 1-octanol | $C_8H_{18}O$ | 130.22 | 56, 41, 43, 55, 70, 69, 84, 83, 29, 31 |
| | 1-pentanol | C5H12O | 88.14 | 42, 56, 41, 70, 31, 29, 57, 43 |
| | 1-hexanol | C6H14O | 102.17 | 56, 43, 41, 55, 31, 27, 29, 69 |
| | 2-methyl-1-butanol | C5H12O | 88.14 | 57, 41, 56, 70, 29 |
| | 3-methyl-1- butanol | C5H12O | 88.14 | 55, 42, 43, 41, 70. 31, 29, 27, 39, 57 |
| | trans-2-hexen-1-ol | C6H12O | 100.15 | 51, 41, 82, 44, 67. 27, 29, 71 |
| | cis-2-penten-1-ol | C5H10O | 83.13 | 57, 41, 39, 44, 29, 58, 67, 55 |
| | 2-octanol | C8H18O | 130.22 | 45, 55 |
| | 1-octen-3-ol | C8H16O | 128.12 | 57, 43, 72, 85, 41, 29, 99 |
| Ketones | 2-butanone | C4H8O | 72.1 | 43, 72, 29, 57 |
| | 2-pentanone | C5H10O | 86.13 | 43, 86, 41, 58, 71, 27 |
| | 1-penten-3-one | C5H8O | 84.11 | 55, 27, 84 |
| | 2-hexanone | C6H12O | 100.15 | 43, 58, 41, 100, 85, 71, 39 |
| | 2-heptanone | C7H14O | 114.18 | 43, 58, 71, 27, 29 |
| | 1-octen-3-one | C8H14O | 126.19 | 55, 70, 27. 43, 41, 39 |
| | 2-octanone | C8H16O | 128.21 | 43, 58, 41, 71, 85 |
| | 2-nonanone | C9H18O | 142.23 | 43, 58, 41, 71, 27, 29, 57, 142 |
| | 4-methyl-penan-2-one | C6H12O | 100.15 | 43, 58, 41, 85, 100, 39, 29 |
| | 6-methyl-5-hepten-2-one | C8H14O | 126.19 | 43, 41, 55, 69, 108, 111, 126, 58 |
| Aldehydes | hexanal | C6H12O | 100.15 | 44, 56, 41, 43, 57, 29, 43, 72, 82 |
| | trans-2-hexen-1-al | C6H10O | 98.14 | 41, 42, 39, 55, 57, 69, 83, 98 |
| | trans-2-pentenal | C5H8O | 84.12 | 55, 84, 83,41, 39, 29, 27, 53, 69 |
| | decanal | C10H20O | 156.26 | 43, 41, 55, 57 29, 70, 68, 82, 112, 95 |
| | trans-2-decenal | C10 H18 O | 154.24 | 43, 41, 29, 55, 70, 83, 57, 39, 27, 98, 110, 136 |

| | | | | |
|---|---|---|---|---|
| | octanal | C8H16O | 128.21 | 43, 56, 44, 41, 84, 57, 55, 29, 27, 69, 82 |
| | trans-2-heptenal | C7H12O | 112.17 | 41, 27, 28, 39, 55, 57, 43, 83, 70 |
| | heptanal | C7H14O | 114.18 | 70, 41, 44,43, 55, 57, 29, 39, 27, 81, 86, 96 |
| | nonanal | C9H18O | 142.24 | 57, 41, 43, 56, 29, 27, 55, 98, 70, 69, 82 |
| | trans-2-octenal | C8H14O | 126.2 | 41, 55, 70, 83, 29, 57, 39, 67 |
| Esters | ethyl acetate | C4H8O2 | 88.11 | 43, 61, 45, 88, 29, 70 |
| | ethyl butyrate | C6H12O2 | 116.16 | 71, 43, 88, 29, 60, 73, 41, 45, 101 |
| | ethyl isovalerate | C7H14O2 | 130.18 | 88, 57, 29, 85, 60, 41 70 |
| | 3-hexenyl acetate | C8H14O2 | 142.2 | 43, 41, 53, 55, 69, 67 |
| | propyl butyrate | C7H14O2 | 130.18 | 43, 71, 27, 89, 41, 60, 39, 29 |
| | hexyl acetate | C8H16O2 | 144.21 | 43, 56, 55, 61, 84, 69, 42, 41, 73 |
| Monoterpenes | limonene | C10H16 | 136.23 | 68, 93, 67, 107, 136, 121, 53, 39 |
| Other compounds | n-octane | C8H18 | 114.23 | 43, 41, 57, 29, 71, 85 |
| | diethyl phtalate | C12H14O4 | 222.24 | 149, 177 |
